# Wide-Detector CT-Based optimized triple Rule-Out CT angiography for emergency chest pain: reducing contrast and radiation without compromising diagnostic quality

**DOI:** 10.1007/s10140-025-02351-4

**Published:** 2025-05-28

**Authors:** Qiuhua Zhang, Kun Wang, Hong Ren

**Affiliations:** 1 Health Information Department, Sir Run Run Shaw Hospital, Zhejiang University School of Medicine, Hangzhou, 310027 China; 2 Department of Radiology, Sir Run Run Shaw Hospital, Zhejiang University School of Medicine, Hangzhou, 310027 China

**Keywords:** Tomography, Triple rule-out, Iodine contrast media, Image quality, Radiation dose

## Abstract

**Background:**

The triple rule-out computed tomography angiography (TRO-CTA) has recently emerged as a technique that noninvasively evaluates the coronary arteries (CAs), the pulmonary arteries (PAs) and the thoracic aorta (TA).

**Objective:**

To evaluate the feasibility of an optimized scanning protocol to reduce the volume of iodine contrast media (ICM), injection rate, and radiation dose in patients undergoing TRO-CTA.

**Methods:**

Patients undergoing TRO-CTA were assigned to either group A or group B using a 16 cm wide-detector CT. Patients in group A were imaged with a traditional triple scanning protocol with a sequence of the PA, CAs, and TA. Patients in group B were imaged using the modified protocol with scanning sequence of PA, TA, and CAs, ICM of 55 ml, and injection rate of 4.5 mL/s. The image quality and effective radiation dose (ED) were compared.

**Results:**

There were no significant differences in basic information between groups A and B. Other than the left PA, RA, and RV, there were no significant differences in the CT attenuation values of relevant vascular structures between groups A and B. There were no significant differences in CNR values between the two groups except the LAD-D and LCX. The image quality scores were comparable between groups A and B except the CAs. However, there were significant differences between the two groups in ICM (*p* < 0.05), scanning time (*p* < 0.001) and ED (*p* = 0. 023).

**Conclusions:**

The optimized TRO-CTA scanning protocol can achieve less ICM and lower ED while maintaining image quality.

## Introduction

Acute chest pain (ACP) is among the most frequent symptoms encountered by emergency room physicians and accounts for more than 6 million visits to emergency departments in the United States annually [1]. The most common causes of ACP include pulmonary embolism, aortic dissection, and acute coronary syndrome [2, 3]. However, ACP is often misdiagnosed. Thus, effective, timely, and accurate identification of the underlying cause is critical to plan an effective treatment strategy.

Triple rule-out computed tomographic angiography (TRO-CTA) is a rapid and non-invasive imaging modality allowing simultaneous acquisition of diagnostic quality images of the pulmonary artery (PA), aorta, and coronary arteries (CAs) to quickly identify the cause of ACP [4, 5]. The current conventional TRO-CTA protocol mostly involves scanning the PA first, then the coronary arteries (CAs), and finally the TA. Although this protocol can achieve a maximum success rate, the time required to inject the iodine contrast medium (ICM) and to obtain an image is relatively long and a large ICM dosage of about 75–130 ml is needed [4, 6–9]. Moreover, a relatively short delay between imaging of the PA and CAs leads to the formation of extensive contrast artifacts in the right atrium (RA) and right ventricle (RV), which seriously inhibits evaluation of lesions of the right CA. However, a longer delay time leads to suboptimal assessment of the thoracic aorta (TA). Thus, a larger dosage of ICM is required to obtain diagnostic quality images of all major vessels. In addition, rapid injection of a large volume of ICM is closely associated with the occurrence of adverse reactions, such as ICM-induced nephropathy and ICM extravasation [10, 11].

Prior studies on TRO-CTA predominantly adopted the PA-CA-TA sequence, where CA imaging is performed shortly after PA scanning (typically within 10 s). This short interval often results in residual high-concentration contrast media in the right atrium (RA) and right ventricle (RV), leading to beam-hardening artifacts that obscure the right coronary artery (RCA). In contrast, our PA-TA-CA protocol inserts a 6-second delay for thoracic aorta (TA) imaging between PA and CA scans. This strategic delay allows partial clearance of contrast media from the right heart, thereby reducing artifacts and improving RCA visualization. To our knowledge, this is the first study to leverage the 16 cm-wide detector CT’s rapid coverage capability to embed TA scanning within the contrast circulation window, achieving both contrast and radiation dose reduction. The advent of 16 cm-wide detector CT systems, such as the GE Revolution™, has revolutionized TRO-CTA by enabling volumetric coverage of the entire heart in a single heartbeat. This technology eliminates the need for multi-phase acquisitions, reduces motion artifacts, and allows precise synchronization of contrast bolus timing—key factors that made the PA-TA-CA protocol feasible.

Therefore, in order to ensure patient safety, the TRO-CTA scanning protocol for ACP was modifiedby adjusting the scanning sequence, where the PA was scanned first, followed by the aorta and finally the CAs. The proposed modified scanning protocol reduced the injection rate and dosage of ICM, while decreasing radiation exposure and maintaining image quality.

Hence, the aim of the present study was to assess the effectiveness of the modified TRO-CTA scanning protocol based on 16 cm-wide detector CT to provide high-quality images for clinical diagnosis, while reducing the injection rate and dosage of the ICM, and limiting radiation exposure.

## Materials and methods

### Study approval

The study protocol was approved by our Institutional Review Board (approval number 2025-2258-01) and was conducted according to the tenets of the Declaration of Helsinki. Due to the retrospective nature of this study, the requirement for informed consent was waived by our Institutional Review Board (approval number 2025-2258-01).

### Study population

**Fig. 1:**
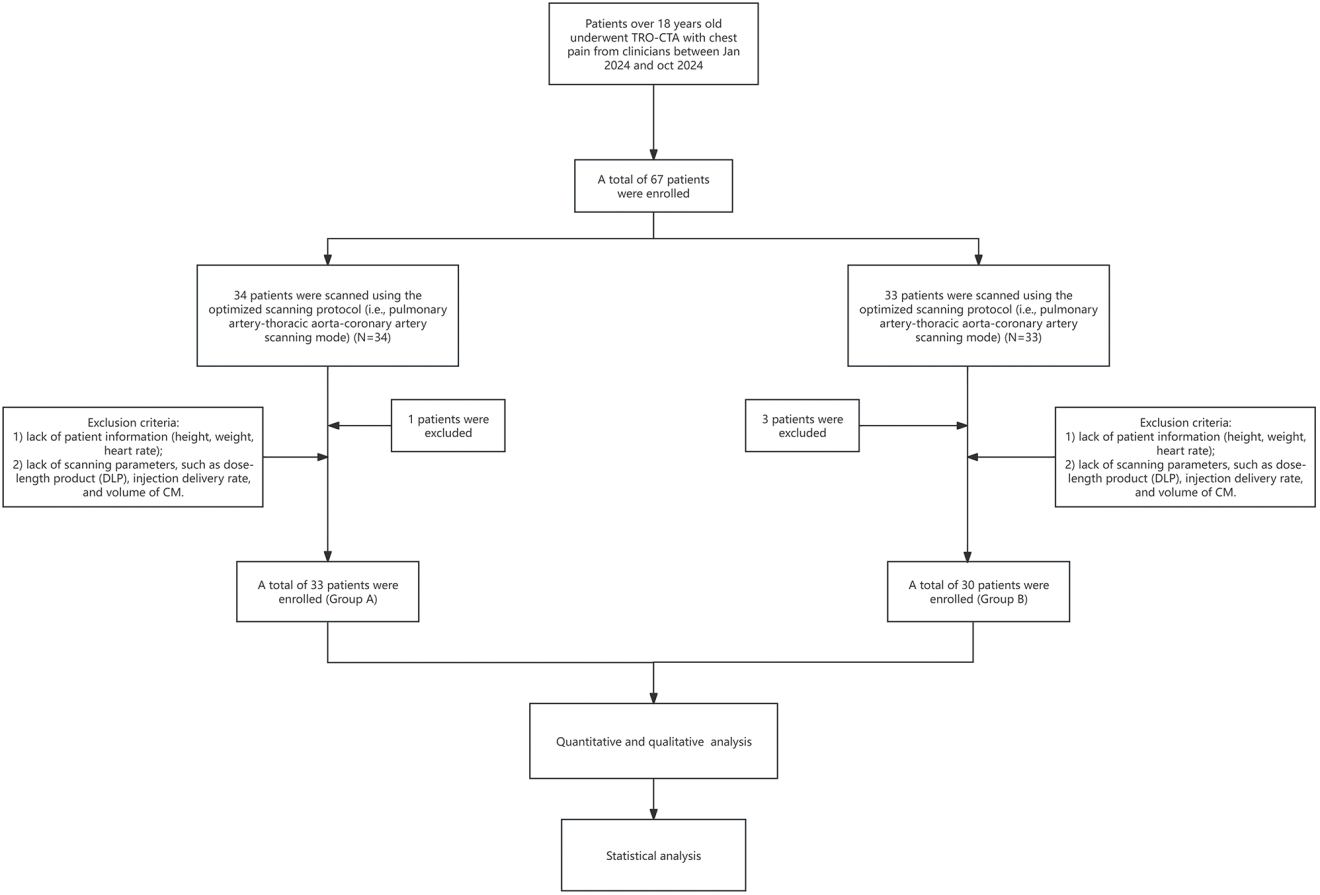
Flow chart of patient enrolment

The study cohort included 67 patients who underwent TRO-CTA for ACP in emergency department of our hospital from January 2024 to October 2024. The inclusion criteria were age ≥ 18 years and a diagnosis of ACP due to pulmonary embolism, aortic dissection, or acute coronary syndrome. The exclusion criteria were as follows: missing basic patient data (i.e., height, weight, and heart rate) or lack of scanning parameters (i.e., dose-length product [DLP], injection delivery rate, and volume of ICM). Finally, 63 patients who successfully underwent TRO-CTA were assigned to either group A (*n* = 33) or group B (*n* = 30) (Fig. 1). Patient assignment to Group A (conventional protocol) or Group B (modified protocol) was based on a threshold date of June 1, 2024. All patients scanned before this date underwent the conventional protocol (Group A, *n* = 33), whereas those scanned after this date received the modified protocol (Group B, *n* = 30). This date-based allocation ensured consistency in protocol implementation during the study period (January–October 2024).

### Image acquisition

All patients underwent TRO-CTA using a Revolution™ CT scanner (GE Healthcare Life Sciences, Chicago, IL, USA) with a 16 cm-wide detector and gantry rotation speed of 0.28 s/rotation. Scans of both groups were conducted with a tube voltage of 100 kV, automatic tube current modulation with the bolus tracking technique, preset noise index of 35 HU, slice thickness of 0.625 mm, and iobitridol concentration of 350 mgI/ml. The scanning modes and ICM injection protocols differed between the groups. No medication was given to the patient prior to the TRO-CTA.

Group A was scanned with the traditional TRO-CTA scanning protocol (PA-CAs-TA). The ROI was also the PA trunk and the threshold was set at 60 HU. Following a delay of 5 s after ICM injection, CT value monitoring of the ROI was performed and following a delay of 3.2 s after automatic triggering, the PA was scanned first (exposure time, 0.7–1.3 s), followed by a 10-s delay prior to an axial scan of the CAs and finally the TA at the shortest interval. Both the PA and TA were scanned by spiral CT with a detector width of 8 cm, pitch of 0.992:1, and exposure time of approximately 1.2 s. The CAs were imaged using a prospective electrocardiogram-triggered scan with a single heartbeat acquisition, detector width of 16 cm, and acquisition window that was automatically adjusted according to the patient’s electrocardiogram. The injection protocol was 75 ml of ICM in the first phase and 40 ml of saline in the second phase to flush, with an injection rate of 5 ml/s in both phases and a total injection time of 23 s. The ICM protocol for Group B followed historical institutional standards (75 ml at 5 ml/s). However, we acknowledge that this fixed-dose approach may exceed the needs of lighter patients. Future protocols should adopt weight-based dosing (e.g., 0.8–1.0 ml/kg).

Group B was scanned with the modified TRO-CTA scanning protocol (i.e., PA-TA-CAs). The region of interest (ROI) was the PA trunk and the threshold was set at 60 HU. CT value monitoring of the ROI was performed with a 2-s delay after ICM injection and a 3.5-s delay after automatic triggering. The PA was first scanned by spiral CT (exposure time, 0.7–1.3 s), followed by a 6-s delay prior to a spiral scan of the TA from the thoracic inlet to the lower edge of the rib-diaphragm angle (exposure time, 1.0–1.3 s), and finally a prospective electrocardiogram-triggered scan with a single heartbeat acquisition of the heart at the fastest scan interval of approximately 3.0–3.5 s (exposure time, 0.3–0.7 s). The total mean scan time was 17–19 s. The PA and TA were scanned with a detector width of 8 cm and pitch of 0.992:1. The acquisition window was automatically adjusted based on the patient’s electrocardiogram during coronary artery acquisition. The injection protocol was 55 mL of ICM in the first phase and 40 mL of saline in the second phase to flush, with an injection rate of 4.5 mL/s in both phases and a total injection time of 21.1 s.

In this study, the Revolution™ CT scanner was used to image the entire heart in one cardiac cycle. Coronary artery images for clinical diagnosis were obtained with free breathing but without control of heart rate.

### Image reconstruction and analysis

The Adaptive Statistical Iterative Reconstruction-Veo algorithm (ASIR-V; GE Healthcare) was used to reconstruct all images with a reconstruction strength of 40%. The SmartPhase technique was used to automatically select the optimal cardiac phase to generate coronary images, and correction of cardiac motion artifacts was performed using the SnapShot Freeze image reconstruction technique.

The CT attenuation value and standard deviation (SD) at the pulmonary trunk, left PA, right PA, aortic root, aortic arch, descending aorta, proximal left main CA, distal left anterior descending artery, distal left circumflex branch, proximal right coronary artery, distal right coronary artery, RA, RV, erector spinae muscle (ESM), and perivascular adipose tissue (PVAT) were measured on axial images on a post-processing workstation. During the measurement, the ROI was set to 20–400 mm^2^ and the vascular wall and areas of calcified plaque were avoided as much as possible. The contrast-to-noise ratio (CNR) of each vessel segment was calculated according to the formula

$$CNR = \frac{{Vessel\,HU - Muscle\,HU}}{{Muscle\,SD}}$$

Image quality was independently assessed by two radiologists blinded to the imaging protocol and with 8 and > 15 years of experience in cardiovascular imaging. The image quality score of the CAs was based on the 15-segment modified classification system of the American Heart Association [12]. A 4-point Likert scoring system was used to assess image quality as follows: 1 point, all major vessels (aorta, PA, and CAs) are clearly displayed with adequate significant luminal enhancement and well-contrasted with surrounding tissues without artifacts; 2 points, all major vessels are well displayed with mild artifacts in the wall or mildly blurred margins and fair contrast with the surrounding tissues; 3 points, all major vessels have moderate or stepped artifacts in the wall with poor contrast but diagnostic; and 4 points, all major vessels have severe motion artifacts in the wall and the vessels appear misaligned and discontinuous, rendering a diagnosis impossible [13].

### Radiation dose

The DLP and volume CT dose index of TRO-CTA imaging for each patient were recorded, and the effective radiation dose (ED) was calculated according to the formula ED = κ* DLP, where κ = 0.014 mSv / (mGy × cm) [14].

### Statistical analysis

Normally distributed continuous data are expressed as the mean ± SD, whereas non-normally distributed continuous data are expressed as the median with interquartile range. The count data are expressed as absolute values and corresponding frequencies/percentages. Statistical analysis between two independent samples was performed with the independent sample *t*-test or Mann–Whitney U-test. The chi-square test was used for statistical analysis of count data. The consistency of the subjective scores of the two physicians was tested using the kappa value, where 0.21–0.40 indicates poor consistency, 0.41–0.60 indicates moderate consistency, and 0.61–0.80 indicates good consistency. A probability (*p*) value of < 0.05 was considered statistically significant. All statistical analyses were performed using IBM SPSS Statistics for Windows, version 26.0. (IBM Corporation, Armonk, NY, USA).

## Results

In total, 63 patients successfully underwent TRO-CTA, which included 33 patients in group A and 30 patients in group B. There were no statistically significant differences in age, height, weight, body mass index, heart rate, gender, or clinical history between the two groups (*p* > 0.05). In addition, there was no significant difference in the final diagnosis between the two groups, as determined by two experienced radiologists (Table 1). TRO-CTA for all patients satisfies diagnostic requirements.

**Table 1:** Patients characteristics

	Group A	Group B	*p* value
Basic information			
Average age (years)	68.1 ± 9.0	64.1 ± 12.1	0.137
Female	16 (53.3%)	19 (63.3%)	0.236
Height (m)	1.65 ± 0.08	1.63 ± 0.07	0.315
Weight (kg)	64.49 ± 10.92	62.57 ± 7.66	0.426
Body mass index (kg/m^2^)	23.67 ± 3.23	23.54 ± 1.82	0.834
Mean heart rate (bpm)	73.27 ± 11.33	71.47 ± 9.88	0.505
**Clinical history**			
Hypertension	17	17	0.682
Coronary artery disease	15	19	0.155
Hyperlipidemia	13	12	0.961
Smoking	9	3	0.081
Diabetes	12	7	0.260
PCI	6	1	0.061
**Clinical outcomes**			
Pulmonary embolism	0	1	
Pneumonia	3	5	
Aortic enlargement	3	0	
Aortic dissection	0	0	
Atherosclerotic ulcer	27	26	
Coronary artery stenosis	20	24	
Myocardial bridge	17	14	
anomalous coronary artery	0	2	
Saline (ml)	40	40	
Saline flow rate (ml/s)	5	4.5	

PCI, Percutaneous Transluminal Coronary Intervention

Except for the left PA, RA, and RV, there were no significant differences in the CT attenuation values of relevant vascular structures including the pulmonary trunk, right PA, aortic root, aortic arch, descending aorta, proximal left main CA, distal left anterior descending artery, distal left circumflex branch, right coronary artery, or ESM between groups A and B (*p* > 0.05). Other than the distal left anterior descending artery and distal left circumflex branch, there were no significant differences in the CNR values of most vessels between the two groups (*p* > 0.05) (Table 2). The image quality scores were comparable between groups A and B for all vessels, with the exception of the CAs. Inter-rater reliability of the qualitative image score was good between the two radiologists (κ = 0.86). All acquired scans met diagnostic criteria (quality score ≤ 3), with no non-diagnostic studies (score = 4) observed in either group.

**Table 2:** Results of the objective and subjective quality analysis

	Group A	Group B	*p* value
**CT Value (HU)**			
PT	553.68 ± 111.88	517.59 ± 73.46	0.140
LPA	529.48 ± 104.32	484.28 ± 67.60	0.048
RPA	525.37 ± 108.90	486.19 ± 68.48	0.096
AO	416.21 ± 105.40	444.88 ± 54.34	0.186
AA	426.98 ± 109.15	427.12 ± 55.22	0.995
DA	444.66 ± 116.17	406.30 ± 63.12	0.114
LMCA-P	457.76 ± 105.43	443.91 ± 91.85	0.582
LAD-D	344.05 ± 74.06	374.14 ± 86.40	0.142
LCX-D	337.58 ± 95.40	368.44 ± 97.01	0.208
RCA-P	434.19 ± 95.81	416.12 ± 87.04	0.438
RCA-D	400.50 ± 111.89	412.81 ± 96.48	0.643
RA	204.87 ± 85.60	117.71 ± 18.16	< 0.001
RV	266.83 ± 134.39	107.33 ± 43.22	< 0.001
ESM	57.77 ± 12.83	58.71 ± 12.19	0.768
PVAT	-120.61 ± 29.10	-111.78 ± 26.74	0.217
**Contrast-Noise-Ratio**			
PT	32.18 ± 11.98	31.48 ± 12.57	0.823
LPA	30.74 ± 11.88	29.26 ± 11.88	0.623
RPA	30.48 ± 11.97	29.49 ± 12.33	0.748
AO	23.36 ± 10.20	26.38 ± 9.85	0.238
AA	24.02 ± 10.69	25.22 ± 9.73	0.644
DA	25.15 ± 11.13	23.83 ± 9.64	0.618
LMCA-P	45.01 ± 17.95	52.00 ± 20.43	0.153
LAD-D	35.86 ± 13.57	45.58 ± 18.93	0.022
LCX-D	34.97 ± 13.01	45.32 ± 20.49	0.019
RCA-P	43.09 ± 16.84	49.53 ± 20.43	0.176
RCA-D	40.27 ± 16.20	49.01 ± 19.66	0.058
**Qualitative image score (4-point scale)**			
Pulmonary artery	2.0 (2.0, 3.0)	2.0 (2.0, 2.0)	0.344
Aorta	2.0 (2.0, 3.0)	2.0 (2.0, 2.0)	0.753
Coronary	3.0 (2.0, 3.0)	2.0 (2.0, 3.0)	0.036

PT, Pulmonary Trunk; LPA, Left Pulmonary Artery; RPA, Right Pulmonary Artery; AO, Aortic Root; AA, Aortic Arch; DA, Descending Aorta; LMCA-P: Proximal Left Main Coronary Artery; LAD-D, Distal Left Anterior Descending; LCX-D, Distal Left Circumflex; RCA-P, Proximal Right Coronary Artery; RCA-D, Distal Coronary Right Artery; RA: Right Atrium; RV: Right Ventricle; ESM, Erector Spinae Muscle; PVAT, Perivascular Adipose Tissue

However, the total ICM dosage and injection rate were significantly lower in group B as compared to group A (55 vs. 75 mL and 4.5 vs. 5.0 mL/s, respectively; Table 3). The ICM dosage was reduced by 26. 7% using the modified scanning protocol (group B). Meanwhile, there was a significant difference in the total scanning time between group A and group B (21.89 ± 3.02 vs. 14.33 ± 0.09s, respectively, *p* < 0.001; Table 3). At the same time, the total ED was significantly lower in group B than group A (6.74 ± 0.73 vs. 7.61 ± 1.90 mSv, respectively, *p* = 0.023; Table 3). The reduction in effective dose (ED: 12%, 6.7 vs. 7.6 mSv) and CTDIvol (4.21 vs. 4.66 mGy for PA/TA scans) between groups, despite identical 100 kVp settings, primarily resulted from faster scan coverage (enabled by the 16 cm-wide detector for coronary imaging) and noise-index-driven tube current modulation. The optimized protocol’s shorter scan time (14.3 vs. 21.9 s) combined with ASIR-V iterative reconstruction allowed lower tube current while maintaining image noise, collectively reducing radiation exposure.

**Table 3:** Comparison of radiation dose parameters

Parameters	Group A	Group B	*p* value
**CTDIvol (mGy)**			
For pulmonary	4.66 ± 1.31	4.21 ± 0.62	0.099
For aorta	4.67 ± 1.31	4.22 ± 0.63	0.090
For coronary	18.17 ± 5.80	17.36 ± 1.88	0.469
**DLP (mSv-cm)**			
For pulmonary	142.42 ± 46.67	118.69 ± 19.80	0.012
For aorta	146.61 ± 40.94	118.80 ± 19.77	0.001
For coronary	254.26 ± 82.99	244.16 ± 28.22	0.529
**ED (mSv)**	7.61 ± 1.90	6.74 ± 0.73	0.023
Total scanning time (s)	21.89 ± 4.38	14.33 ± 0.09	< 0.001
Contrast media (ml)	75 ± 0	55 ± 0	N/A
Injection rate (ml/s)	5.0 ± 0	4.5 ± 0	N/A

CTDIvol, volume CT dose index; DLP, Dose-Length Product; ED, effective dose

## Discussion

The prevalence of ACP in the general population is approximately 25% [15]. The number of emergency room visits for ACP in the United States exceeds 6 million annually, accounting for the second most common cause of visits by adults to an emergency room [16]. The TRO-CTA scanning protocol allows simultaneous acquisition of images of all major vessels with a single injection of ICM, thereby shortening the diagnostic time and providing rapid identification of the cause of ACP. In this study, tube voltage (100 kVp) was used in the scanning protocol of both groups, which significantly reduced the radiation dose for group A as compared to group B (7.61 ± 1.90 vs. 6.74 ± 0.73 mSv, respectively) and was significantly lower than the radiation dose reported in previous studies of TRO-CTA (approximately 9.1 mSv) [13].

**Fig. 2:**
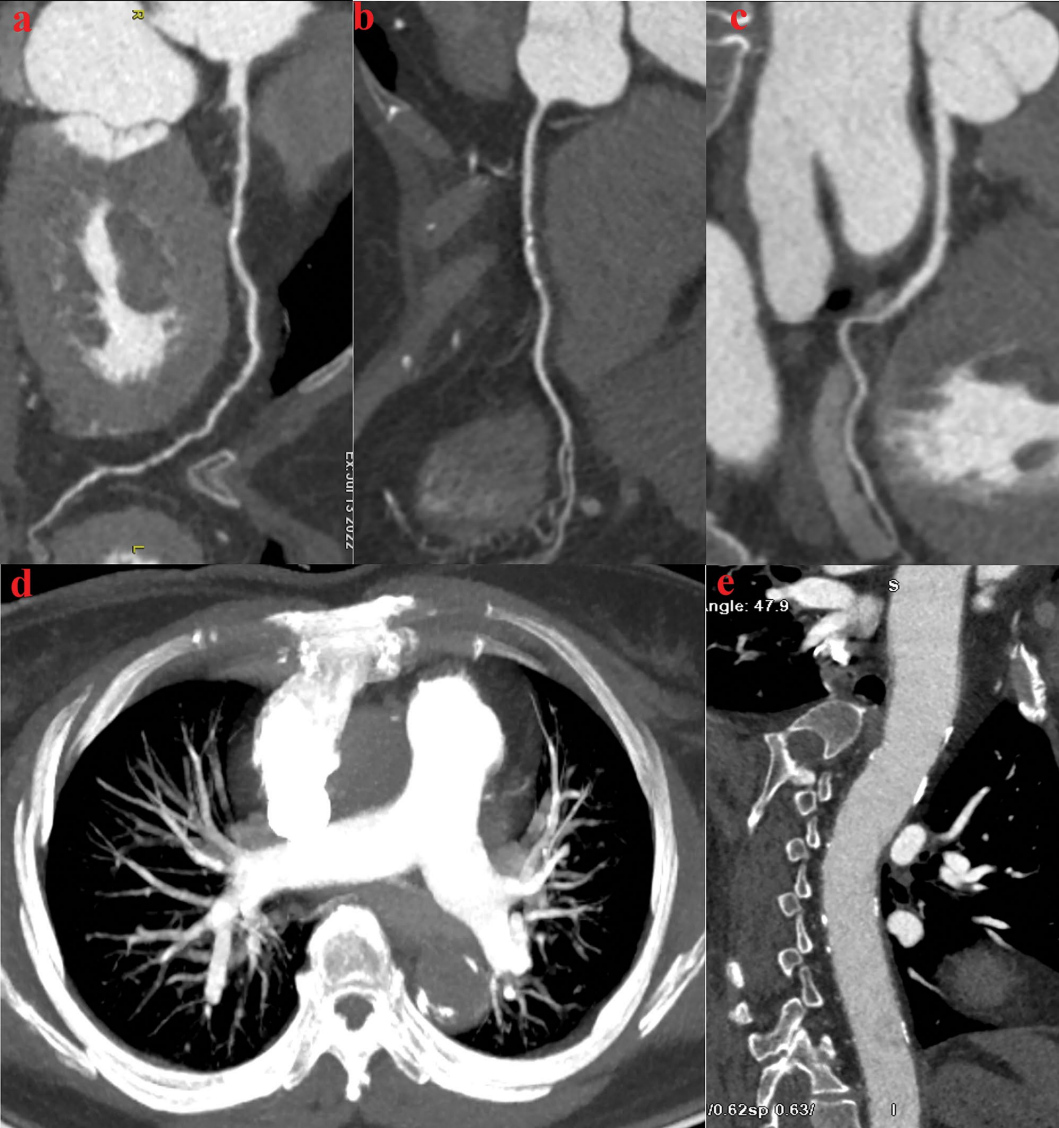
60-year-old woman with chief complaint of chest pain (heart rate: 68 bpm). Images were acquired with the optimized TRO-CTA scanning scheme. Curved multiplanar reformat CT images show the left anterior descending artery (**a**), right coronary artery (**b**), left circumflex artery (**c**) and the thoracic aorta (**e**). The maximum intensity projection (MIP) images (**d**) show the pulmonary artery

The choice of scanning mode is an important pre-condition for effective and rapid examinations. An optimized scanning protocol not only provides high-quality imaging data for clinical purposes (Fig. 2) but also shortens the examination time and increases efficiency. The coronary vessels are thin and curved, and the optimal peak time for iodine contrast is about 14–28 s [17]. In contrast, the conventional scan mode emphasizes enhancement of the coronary vessels and the TA is scanned last. So, longer time periods are needed to maintain the contrast and complete the entire examination. In addition, a larger volume ICM is used. In this study, imaging of the TA with the Revolution™ CT scanner was completed within the waiting time for the ICM to reach a peak value, thereby meeting the optimal time for scanning both the TA (6–10 s) [18]and CAs (14–17 s). High-quality images of each coronary vessel were obtained within a shorter time period to complete the full scan in about 5–7 s as compared to conventional imaging.

Although there were no significant differences in the CT values of any of the coronary vessels in this study, differences in the subjective scores of coronary image quality between the two groups were statistically significant, possibly due to the radiographic artifacts formed by residual ICM in the RA and RV during the coronary scan, which reduced the difference in enhancement between the vessels and surrounding tissue and to some extent, observation of the RCA by the radiologists. While CNR can effectively indicate differences in enhancement between vessels and surrounding tissues [19], the CNR values of all segments of the coronary vessels were higher in group B than group A, indicating that the optimized scanning protocol effectively reduced the influence of artifacts of the RA and RV on coronary image quality and improved contrast between the coronary vessel lumen and surrounding tissues, resulting in a significantly better mean subjective score of coronary image quality in group B as compared to group A (1.93 vs. 2.53, respectively).

The optimized scanning protocol improved image quality of all major vessels and effectively reduced the amount of ICM, which was positively correlated with the incidence of adverse reactions, such as acute kidney injury. In this study, the amount of ICM was reduced by 26.7% in group B as compared to group A (55 vs. 75 ml, respectively), which was favorable as compared to 80 ml reported by Burris et al. [13]and 90 ml reported by Mohamed et al. [7] This reduction was enabled by synchronizing the TA scan with the contrast peak waiting period, a strategy uniquely feasible on wide-detector CT systems. Notably, the 55 ml ICM dose in Group A equates to 0.85 ml/kg for a 65 kg patient, aligning with the ESC 2023 recommendation for CIN prevention [20]. This dose is 31% lower than the conventional 75 ml regimen, potentially benefiting patients with renal impairment. In addition, the time to complete the examination was approximately 14 s in group B, as compared to 21.1 s in group A, suggesting that the shorter time to complete the full scan and time for ICM injection could further reduce the volume of ICM from 55 ml.

Some limitations to this study should be addressed. First, this was a single-center study. Thus, a multi-center study is required to confirm the results. Second, the ICM injection protocol was not individually tailored to the patient’s weight, body mass index, or heart rate. Third, the study population had a mean BMI of 23.6 kg/m², with no explicit exclusion criteria based on body weight. The relative scarcity of obese patients (BMI ≥ 30 kg/m²) in our cohort reflects the demographic characteristics of the emergency department population during the study period, rather than intentional exclusion. While our cohort’s mean BMI (23.6 kg/m²) reflects the demographic profile of our emergency department, we recognize that this limits generalizability to populations with higher BMI (e.g., U.S. average: 29–30 kg/m²). Future studies should validate this protocol in obese patients, adjusting tube voltage (e.g., 120 kVp) and ICM dose proportionally to weight.Finally, sensitivity and specificity for coronary diagnosis were not compared with the gold standard DSA.

In conclusion, the optimized scanning protocol in this study achieved a “double low” mode by using less ICM and a lower radiation dose for all examinations. The protocol also reduced the impact of contrast artifacts on the quality of coronary images of the RA and RV, while providing high-quality images of the vessels to improve clinical diagnosis.

## Data Availability

All data generated or analysed during this study are included in this published article.
